# Human microRNA-299-3p decreases invasive behavior of cancer cells by downregulation of Oct4 expression and causes apoptosis

**DOI:** 10.1371/journal.pone.0174912

**Published:** 2017-04-20

**Authors:** Axel R. Göhring, Stefanie Reuter, Joachim H. Clement, Xinlai Cheng, Jannick Theobald, Stefan Wölfl, Ralf Mrowka

**Affiliations:** 1 Experimentelle Nephrologie, Klinik für Innere Medizin III, Universitätsklinikum Jena, Jena, Germany; 2 Abteilung Hämatologie und Internistische Onkologie, Klinik für Innere Medizin II, Universitätsklinikum Jena, Jena, Germany; 3 Institut für Pharmazie und Molekulare Biotechnologie, Abteilung pharmazeutische Biologie, Universität Heidelberg, Heidelberg, Germany; University of Hong Kong, HONG KONG

## Abstract

**Purpose:**

Oct4 was reported to be one of the most important pluripotency transcription factors in the biology of stem cells including cancer stem cells, and progressed malignant cells. Here we report the investigation of gene expression control of Oct4 by selected human microRNAs and the physiological effect of Oct4 silencing in invasive cancer cells.

**Methods and results:**

High throughput luciferase activity assay revealed the microRNA-299-3p to be the most effective in reducing gene expression of Oct4, which was confirmed by Western blot analysis and Oct4 promoter activity in a target luciferase assay. Furthermore, it could be demonstrated that downregulation of Oct4 by microRNAs-299-3p in breast cancer and fibrosarcoma cells lead to a decreased invasiveness in a microfluidic chip assay. Additionally, microRNA-299-3p causes apoptosis in cancer cells. Comparison with Oct4 specific siRNA transfection confirmed that this effect is primary due to the blockade of Oct4 expression.

**Conclusion:**

The results suggest that microRNA-299-3p is an interesting target for potential clinical use. It may be able to decrease invasive behaviour of carcinoma cells; or even kill these cells by causing apoptosis.

## Introduction

Stem cell genes like Oct4 (*Octamer-binding factor*) are known for maintaining the pluripotency state of embryonic and induced stem cells [1] as well as cancer cells [2], [3]. Understanding the genetic regulation of stem cell genes thus might lead to novel technologies for stem cell and cancer therapy.

MicroRNAs are important for the regulation of gene expression and were discovered in 1993 [4]. They are evolutionarily highly conserved; and are usually processed into 21–23 bases long short non-coding nucleotide sequences and occur in both invertebrates and vertebrates [5]. They regulate target gene expression by hybridization to partially complementary sequences in the 3' untranslated region (3’UTR) of mRNA [6] and block translation [7] or lead to degradation of the mRNA [8]

Based on bioinformatic predictions, up to 30% of all human genes could be regulated by microRNAs [9]. The genes encoding microRNAs are located either within protein-encoding genes or in separate loci of the genome [5], [10]. Since microRNAs are implicated in the specification of many cell types showing cell type specific expression, microRNAs represent an important research topic in the cell differentiation and stem cell research. For example, the miRNA clusters 290–295 [11], [12], 302 and 17–92 [13] were identified as stem cell character-sustaining microRNAs. Therefore, they are interesting for the production of induced pluripotent stem cells (iPS). Human microRNA-145 and the miRNA-290 cluster were described to repress expression of Oct4, Sox2, and Klf4 genes and consequently had been described as differentiation-promoting [14]. Apart from stem cell biology, “stemness” characteristics also have been found in mammalian cancer cells, and it is assumed that every malignant tumour entity contains stem cell-like cells [15]. These cancer stem cells can mediate resistance to chemotherapeutic agents, e.g. in prostate carcinomas [16]. Therefore, microRNAs, which selectively target and repress the factors required to maintain cancer stem cells, could serve as therapeutic agents in the future [17]. In this study, a systematic high throughput assay was performed to identify human microRNAs, which target the 3’UTRs (*untranslated region*) of Oct4 transcripts, using a commercially available library of 477 mature human microRNAs (Ambion, life technologies). Sequences of selected top hits were mapped to potential target sites in the 3’UTR of Oct4. Furthermore, we examined physiological effects of the most effective microRNAs in cancer cells.

## Materials and methods

### Cloning of Oct4–3’ UTR vector

The 3 'UTR sequence of Oct4 was synthesized by PCR from a human genomic template. This sequence was cloned into a fusion plasmid with the reporter genes for Firefly and Renilla luciferase controlled by CMV promoter. The fusion plasmid called “pc5/Psi” was cloned previously by using parts of pcDNA5/FRT (Invitrogen) and psiCHECK-2 (Promega, catalog-no. C8021). Renilla luciferase was coupled with the 3’UTR serving as reporter gene, and Firefly luciferase served as cell number control.

### Stable transfection of HEK293-FRT cells by homologous recombination

Using the *Flp-In* system (Invitrogen), HEK293-Flp-In cells were transfected with the 3'UTR dual luciferase vectors. The cells were bought directly from the company. Both the cells and the plasmids possessed a Flippase Recognition Target site (FRT). The Flippase gene was provided by an additional vector called pOG44 (Invitrogen). The enzyme recognizes the FRTs, cuts the DNA and ligates the 3’UTR vectors with the genomic site. The resulting transgenic HEK293 cells were thus isogenic and could be selected by hygromycin due to the resistance gene of the vector. The cellular genomic transgene was proved by PCR.For the preparation of the transfection solution 100 μl of Opti-MEM (Gibco), 2 μg of the pc5/Psi vector and 18 μg of pOG44 were mixed. Further 100 μl of Opti-MEM were mixed with 10 μl Roti-Fect (Roth). Both solutions were united and incubated for at least 15 min. at ambient temperature. After 24 hours, the transfection medium was replaced by fresh complete medium (DMEM based, Gibco) and the cells were cultured for another day. This was followed by a cell splitting 1: 5. After growth of the cells, 10 ml of complete medium with 300 μg / ml hygromycin B were then added to the cells. After 24 h, the medium was replaced by 10 ml complete medium supplemented with 100 μg / ml hygromycin. The cells were further cultured for at least a week.

### microRNA and Oct4 interaction 3’UTR and validation

The microRNA library contained 477 individual human microRNAs distributed on six 96-well plates (Ambion, Pre-miR microRNA Precursor library-human V3, Cat.4385830). Hint: The manufacturer’s term *pre-microRNA* (double stranded DNA w/o stem-loop structure) must not be confused with the scientific concept (stem loop DNA). The absolute amount per miRNA species was 250 pmol. Using a multichannel pipette, the nucleotides were dissolved in 50 μl RNAse-free water to achieve a concentration of 5 pmol/ μl. The plates were then cryopreserved (-20°C). In preparation for the transfection, the miRNA solutions were dispensed into luminometer plates (Greiner; 3 pmol/ 5 μl) using a cell culture robot (CyBio Selma) under sterile conditions. For the transfection, the plates were thawed and centrifuged briefly to collect all the liquid in the ground. Using the luminometer (Labsystems), 15 μl of transfection solution (14.8 μl of Opti-MEM, 0.2 μl Lipofectamine RNAiMAX) were injected into each well. The plates were incubated for at least 15 min at 20°C in the dark in order to achieve a complete complexation of liposomes and nucleic acids. Then 100 μl of cell suspension containing 12,500 cells were injected into each well with the luminometer after previous sterilization of the injector hoses. After incubation of the plates for 24 h at 37°C and 5% CO_2_ the cells were lysed with 20 μl of 1:5 diluted passive lysis buffer (Promega) and shaken well. In order to perform the luciferase assay later, the plates were frozen at -20°C. Not later than three days after cryopreservation, the luciferase activity in the cell lysates was determined using 100 μl ambiently temperated Firefly and Renilla buffer each. The luminometer was programmed to measure with a delay of six seconds after injection and a duration of ten seconds. The obtained luminescence values were standardized using a z-transformation to make the signals of all the samples comparable. This standardization relates the mean and standard deviation of the entire 96-well luminometer plate values. The z-value triplicates were arithmetically averaged and compared by ranking.

### Interaction analyses of the miRNA interaction and Oct4

**Fig 1:**
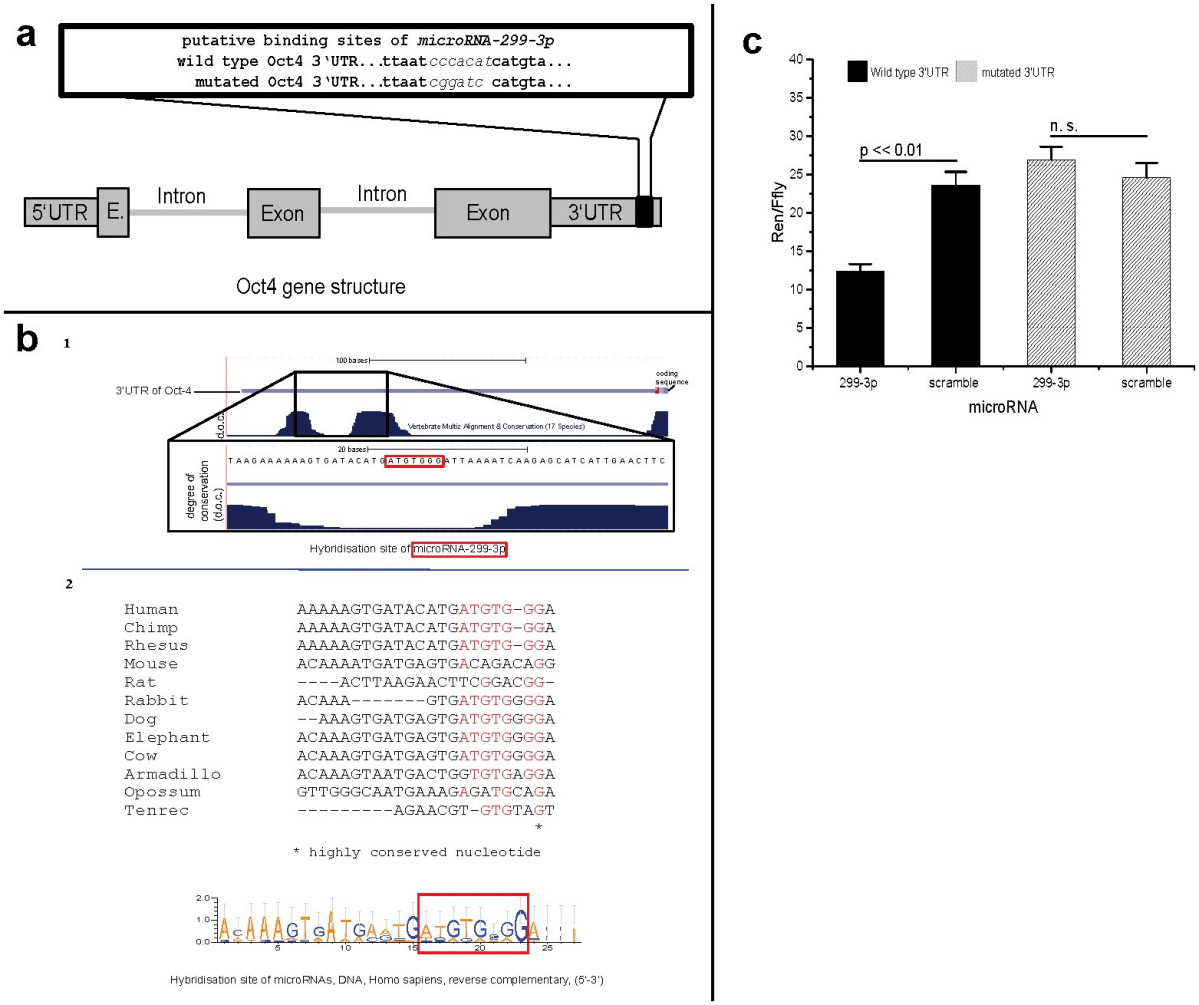
Binding sites of microRNA-299-3p in 3’UTR of Oct4. **a: Wild type and mutated sequence of Oct4 3‘UTR**. Mutation was used for verification and mapping of putative microRNA target sequence in a luciferase reporter assay. Mutated nucleotides are *in italics*. **b: Evolutionary conservation of microRNA-299-3pbinding sites in human Oct4 gene on chromosome 6**. Site for 299-3p is poorly conserved. (1) Degree of conservation based on alignment of human sequence with those of 17 other vertebrates obtained from UCSC server (http://genome.ucsc.edu/; hg18 algorithm) (2) Illustration of alignment results by ClustalW2 algorithm of human sequence with eleven other mammals. Size of colored base symbols refer to degree of conservation. Image was created by weblogo server (http://weblogo.berkeley.edu/). **c: Mutation of putative binding site of Oct4 3´UTR inhibits down-regulating effect of microRNA-299-3p in comparison to wild type sequence**. Scramble: non-human miRNA sequence (negative control). Error bars indicated as SEM. Non-parametric ANOVA (SRH-test): p < 0.01. Single p-values of interaction significance tests are indicated in the graph. Effects of 3’UTR: p = 0.0006 and effect of microRNA: p = 0.095 and the UTR specific miRNA influence (statistical interaction): p = 0.0017. Pair-wise comparisons and their p-values are indicated in the figure.

To investigate what area of the Oct4 3 'UTR is bound by effective microRNAs (miR299-3p), the putative binding sites were determined using the bioinformatical service TargetScan (www.targetscan.org).The potentially hybridizing nucleotides for both microRNAs were partially replaced in silico (Fig 1a). The two mutated 3'UTR sequences with additional restriction sites were synthesized by a service provider and afterwards cloned into the pc5/Psi dual luciferase vector. The two plasmids with both mutated 3'UTRs of Oct4 and a pc5/Psi vector with an unaltered 3’UTR sequence serving as control were transiently transfected into HEK293 cells as described above (individually ordered microRNA -299-3p; Ambion PM10063 and PM10448). The transfection medium was added to 500,000 cells in a 6-well plate with 2 ml medium per well. After 24 h at 37°C and 5% CO_2_, the cells were trypsinized and seeded in a microtiter plate luminometer (96w) with a concentration of 25,000 cells per well. The cells were transfected with miR-299-3p and negative control. After additional incubation for 24 h, the cells were lysed and the luciferase activity was determined using the luminometer.

### Long-term measurement of miRNA action with lentiviral reporter system

In order to investigate the indirect effect of miR-299-3p on Oct4 targets, a NCC-IT based cell line (DMSZ, Brunswick, Germany, ATCC CRL-2073) with a genomically integrated HIV-derived lentiviral Oct4-reporter construct (Cignal, Qiagen) was transfected with this two microRNAs, and miR-negative control, respectively. The Oct4-reporter construct consists of an Oct4-responsive promoter sequence and the gene for the Firefly luciferase. The gene for Oct4 is expressed natively in the selected cell line [18]. 12,500 cells per well were transfected with 3 pmol miRNA incl. negative control (see above) in 100 μl medium (DMEM w/o phenol red, 10% FCS, 1% HEPES, 250 μM Luciferin D) in a 96-well luminometer plate. The response of NCC-IT-Oct4 cells to miRNA stimulation was recorded over a period of 24h in a temperature-controlled luminometer at 37°C (*Top Count*, Packard). For comparison, the same experimental procedure was used with siRNA against Oct4, and against AGTR1 as control (ThermoFisher Scientific, Silencer Select, s10871 (Oct4) and s1180 (AGTR1)).

### Invasion assays

The invasion assays were performed in hydrophilized thermoplastic microfluidic chips (made of Cyclo-olefin polymer/Zeonor, Fluidik 221, microfluidic ChipShop). The chamber of a chip was filled half with 50μl Matrigel (BD) containing the fluorescent dye DY-630-OH (c = 100 pg/ μl, Dyomics). After thermosetting of the Matrigel an additional air outlet was created with a glowing felting needle in the middle of the chamber. The day before highly invasive breast carcinoma cells (MDA-MB-231, ACC-732 by DMSZ, via lab JHC and clinic for women’s health, Jena) were transfected with microRNA-299-3p, miR-negative control, siRNA against Oct4 and AGTR1 (150 pmol and 400,000 cells per well in a 6-well cell culture plate, siRNA as above). Then, a cell suspension with a concentration of 1,000 cells /μl was injected into the other half of the chip chambers. After 48 h, the contact area of cell suspension and Matrigel was photographed with a fluorescence microscope (Zeiss Axiocam Observer Z1 with camera Axiocam MRM; software ZENblue).

Cell line HT1080 (ACC-315 by DMSZ, via lab JHC): Because these cells formed an invasion front less accessible for optical analyzis, the cell number was enlarged (3.000 cells/μl), and the contact area of cells and matrigel was already photographed after 24 h.

### Cytotoxicity and apoptosis assays

To analyze microRNA-effects on invasive behavior, we used two highly invasive human cell lines: MDA-MB-231 (breast cancer, see above) and HT-1080 cells (fibrosarcoma, s.a.). To distinguish between cancer specific and general effects of miRNA-299-p, we stimulated an additional non-cancer human cell line, KG-1 (ACC-14, DMSZ, via lab Anita Voigt, University Children’s hospital, Jena; myeloid hematopoietic-like cell line [19]) MDA-MB-231, HT-1080 and KG-1 cells, respectively, were pre-stained with Hoechst 33342 (bisBenzimide, as cell number control) with a concentration of 1 μg/ml dye in full RPMI 1640 medium or DMEM incl. 10% FCS and 1% Pen/Strep (DMEM for KG-1). 24h later the Hoechst 33342 medium was removed and the cells were transfected with 3 pmol miRNA -299-3p, and siRNA against Oct4, respectively, in 96-well luminometer plates as described above. Simultaneously, the cells were stained with CellTox Green dye (Promega) following the manufacturer´s recommendations for endpoint express protocol. After additional 48 h, the fluorescence of CellTox Green (CTG) and Hoechst 33342 (H) was measured in a standard plate reader. The CTG and H fluorescence values of each replicate were used to create a ratio.

Similarly, pre-stained MDA-MB-231, HT-1080 and KG-1 cells, respectively, were transfected, and 24h later Caspase-Glo 3/7 assay (Promega) was used to measure caspase activity according to the manufacturer´s protocol. The Caspase and H fluorescence values of each replicate were used to create a ratio.

### Protein detection

In order to investigate the effect of microRNA-299-3p on the protein level of Oct4, 62,500 NCC-IT-Oct4 cells (see above) were seeded into a 24-well-cell culture plate and were transfected with 0.75 μl Lipofectamine RNAiMAX and 15 pmol microRNA-299-3p and the negative control in Opti-MEM (ad 500 μl).This procedure was repeated every 24 h twice. A degenerative miRNA effect could be observed after 3 days by visual inspection. The cells were lysed in 40 μl urea buffer (6 M). The cell debris was removed by centrifugation. The protein content of the solution was determined using a spectrophotometer. For the detection of the two Oct4 protein isoforms (33–45 kDa), a concentration of 10% was chosen for the polyacrylamide gel separation. Vinculin was used as a loading control. For electrophoresis, 40 μl protein solution (c = 50 μg / ml) were applied. After protein transfer, the blot membrane (PVDF) was cut by using scissors and the two parts were incubated with one of the two primary antibody solutions (against Oct4, isoform A and B, *sc-5279*, 1:500, Santa Cruz; each with antibody against vinculin, *4650*, 1:1000 Cell signaling technology [20]). The blot membrane parts were washed, put together again and incubated with a secondary antibody solution (infrared chromophore, *35568*, Thermo Fisher). The infrared signals of the hybridizing secondary antibodies were detected with the Licor Odyssey 3.0 device and digitized. The signal bands in the files were obtained by densitometry measured with an Image Analyser program (Aida).

## Results and discussion

We used a comprehensive human miRNA library screen in order to analyze the action on the Oct4 3’UTR. For the top candidates further validation experiments were performed and possible microRNA binding sites were predicted and confirmed. The two top candidates were further analyzed with respect to invasion behavior and induction of cell death.

### Action of microRNA on Oct4 3’UTR in a High Throughput Screening assay (HTS) and protein detection

**Fig 2:**
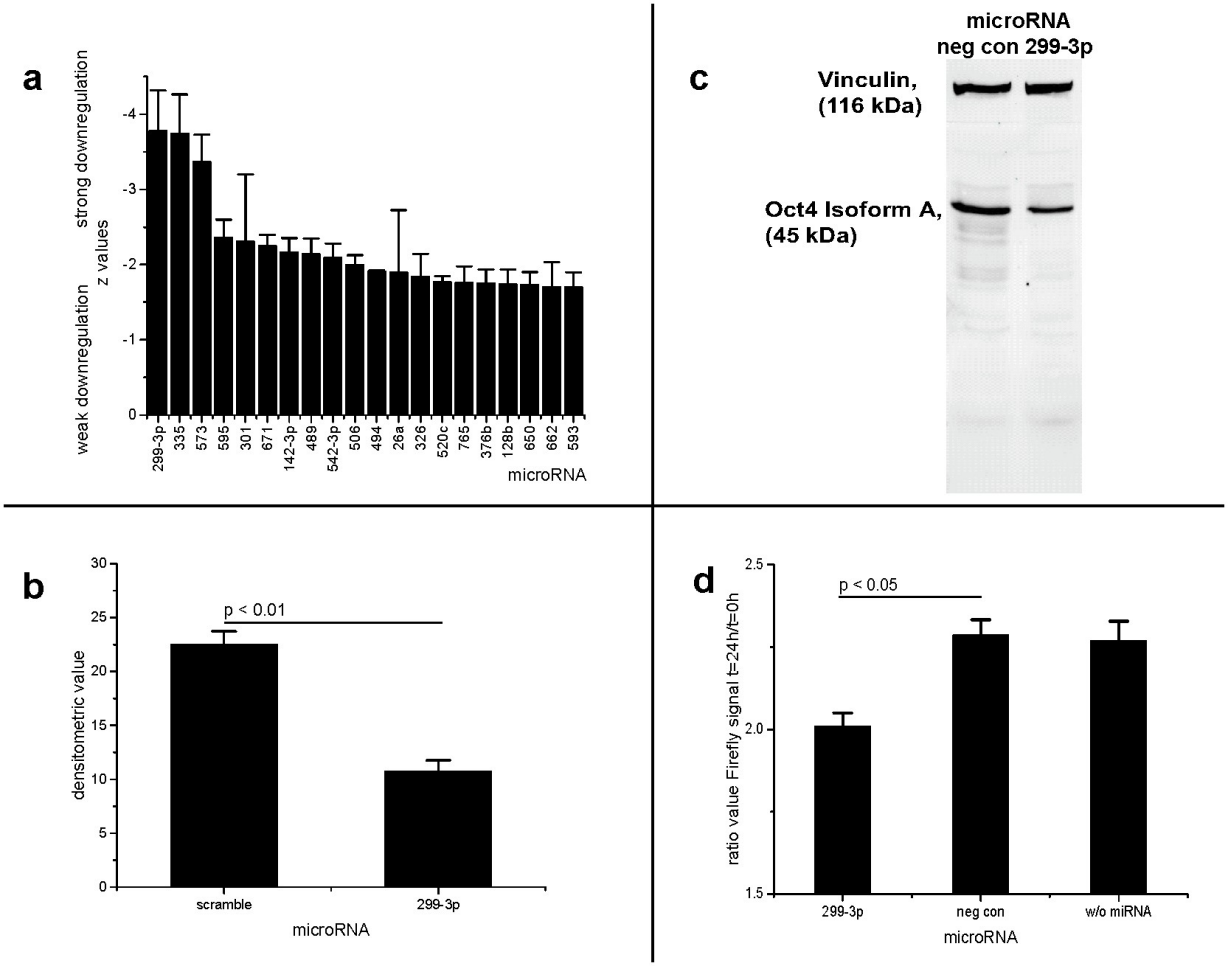
microRNA-299-3p downregulates Oct4 expression. **a: The 20 most effective microRNAs down-regulating Oct4 expression. The micro RNA originated from a library. Values were measured by dual luciferase reporter high throughput assay**. Bars indicate individual z-values (negative values refer to high regulative effect of microRNA). Error bars indicate standard error of the mean (SEM). The most negative z values correspond to the strongest downregulation effect. **b and c: Oct4 protein production is decreased by microRNA-299-3p in NCC-IT cells**. Scramble: non-human miRNA sequence (negative control). b) Oct4 transcription factor, isoform A, infrared signal detected in differentially transfected NCC-IT-Oct4 cells (vinculin as loading control). c) Densitometric detection of Oct4, isoform A, in NCC-IT-Oct4 cells transfected with microRNA-scramble, -299-3p. miR-299-3p significantly downregulates Oct4 synthesis. Error bars indicated as SEM. Non-parametric Kruskal-Wallis-test: p < 0.01. Post-hoc test results (Tukey) indicated in the graph. **d: microRNA-299-3p downregulates promoter activity of Oct4 target**. Luciferase reporter assay measures activity of an Oct4 consensus promoter. Data were obtained over 24h. The mean of values of first and last ten minutes were taken to calculate the ratio. Scramble: non-human microRNA sequence (negative control). Error bars indicate SEM. Non-parametric Kruskal-Wallis-test: p < 0.05. Post-hoc test results (Tukey) indicated in the graph.

Isogenic HEK 293 cells containing a dual luciferase reporter with the 3’UTR of Oct4 were stimulated with 477 microRNA species (triplicate each). According to the luciferase signals, the microRNAs-299-3p was the two most effective molecule for repression of Oct4 (see Fig 2a).

In Western blots the Oct4 protein (isoform A) was quantified in NCC-IT-Oct4 cells which were transfected with microRNA-299-3p (and negative control, respectively, see Fig 2b and 2c).

### Downregulation of promoter activity of Oct4 target by microRNAs

To confirm the results of the HTS, microRNA-299-3p was applied in an additional physiological luciferase assay. To analyze possible Oct4 independent effects of microRNA, the experiment was repeated using Anti-Oct4-siRNA instead of microRNA.

Cells transfected with microRNA-299-3p showed a decrease of Firefly luciferase signal in comparison to controls (see Fig 2d). A similar effect could be observed in cells transfected with Anti-Oct4-siRNA (see S1 Fig). The luciferase activity in living cells was measured over a period of 24 h in a temperated luminometer.

### Mapping of the microRNA interaction site in the Oct4 3’UTR

The predicted hybridization site of microRNA-299-3p was located at base position 200–206 of the Oct4 3’UTR.

The hybridizing nucleotides were compared using a database to their similarity with the sequences of 17 vertebrate species including mammals, birds and fishes (http://genome.ucsc.edu/). It was found that the binding sequence of miR-299-3p is located in an only moderately conserved region (see Fig 1b). On the contrary, the miR-299-3p seems to have evolved its function as a repressor of Oct4 much later at the level of primates. This finding is congruent with the view, that microRNAs are extremely important factors for a divergent development of primate species [21].

In order to validate the bioinformatically predicted hybridization sites of the microRNAs-299-3p, two additional dual luciferase reporter gene vectors with a mutated 3'UTR were generated (see Fig 1a).

In transiently transfected HEK293 cells containing the mutated 3’UTR vector the miRNA-299-3pcould not downregulate the Renilla luciferase anymore.

In contrast, reporter luciferase signal was significantly reduced in the cells with the unaltered sequence of the 3'UTR of Oct4 (see Fig 1c). The bioinformatically predicted hybridization site of miRNA-299-3p could thus be confirmed.

### Invasion assays and cytotoxicity/apoptosis assays

**Fig 3:**
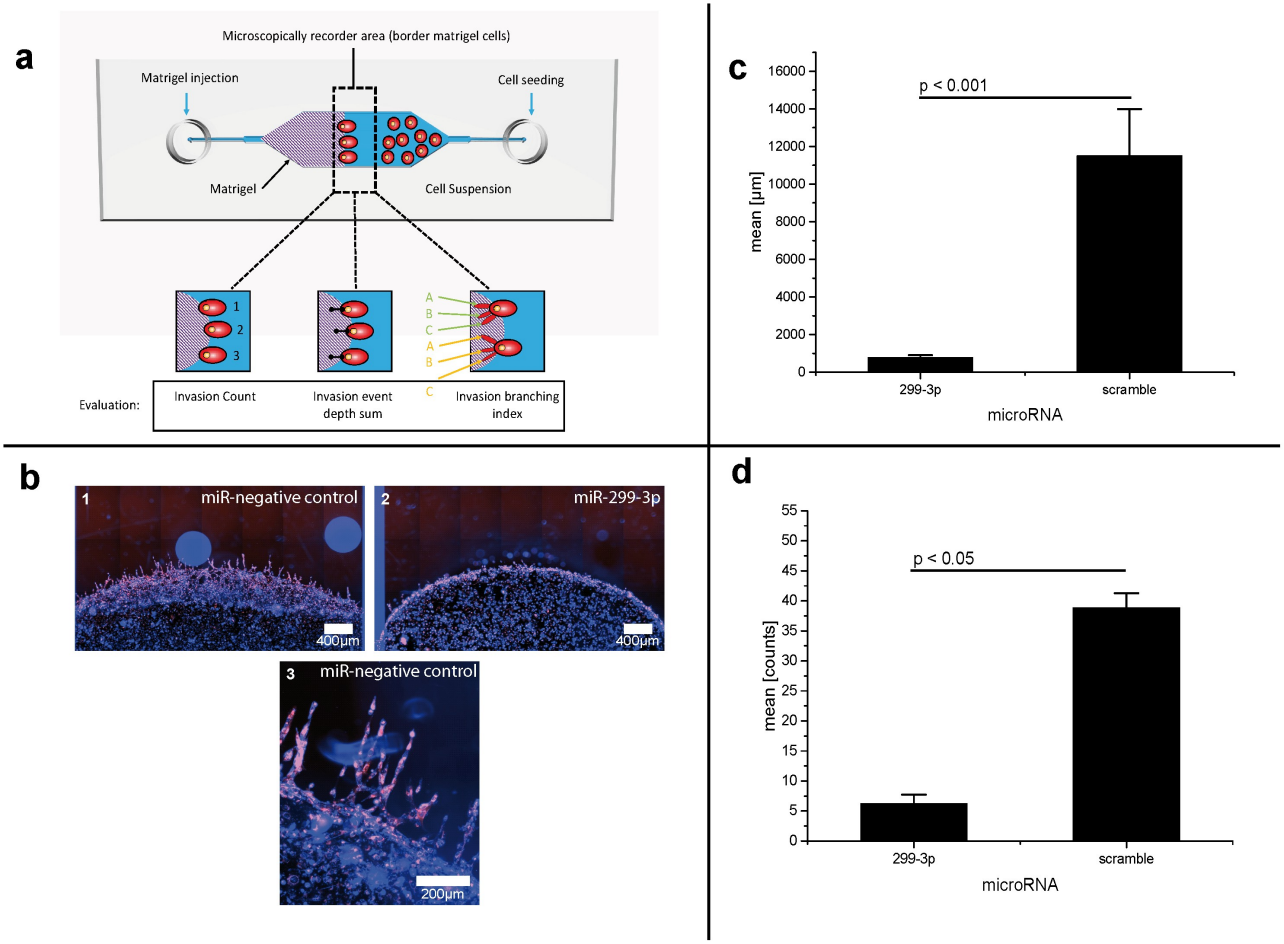
MicroRNAs suppress invasion of mammacarcinoma cells (part I). **a: Schematic setup for evaluation of invasion process in in-house modified microfluidic cell culture chips**. Chips have microscope slide standard size (76 x 26 mm) and consist of Cyclo-olefin-polymer (COP aka Zeonor). Total volume of culture chambers is 100 μl. **b: Decreased invasive behavior of mammacarcinoma cells caused by transfection with microRNA-299-3p(fluorescence sample images)**. MDA-MB-231-cells, transfected with pre-microRNA-scramble (negative control) (1, magnified detail 3), -299-3p (2). Cells with microRNA-299-3pshow an extremely decreased invasive behavior in relation to negative control. False colors image. Object lense: 10x /1,2,; 40x/ 3. **c: microRNA-299-3p decreases invasion distance of MDA-MB-231 breast cancer cells**. Invasion assay, sum of invasion distance in μm. Scramble: non-human miRNA sequence (negative control). Error bars indicated as SEM. Non-parametric Wilcoxon test: p < 0.01. Post-hoc test results (Tukey) indicated in the graph. **d: microRNA-299-3p decreases number of invasion events of MDA-MB-231 breast cancer cells**. Invasion assay, sum of invasion events. Scramble: non-human miRNA sequence (negative control). Statistics: Error bars indicated as SEM. Non-parametric Wilcoxon test: p < 0.01. Post-hoc test results (Tukey) indicated in the graph.

Oct4 has been described as a factor involved in cell invasion [22]. Due to stemness properties of malignant tumor cells, it could be useful to target Oct4 (and other stem cell typic genes like Sox2 or Nanog) in order to down-regulate their invasive behavior. Therefore, we analyzed the human anti-Oct4 microRNA-299-3p and in invasion experiments. For this purpose, a novel microfluidic invasion assay system was established. It is based on the use of cell culture chips combined with a non-toxic fluorescent dye and Matrigel (see Fig 3a). Microfluidic chips are an application of the lab-on-a-chip (LOC) technology that allow the culture of cells in a small volume and represent a recent technology for advanced assays in chemistry [23] and life sciences [24]. The microfluidic LOC technology facilitates automatization of cell culture tasks at considerably lower costs [25]. Some microfluidic devices offer the combination of special chips and hydrogels as invasion matrix [26]. Such culture chips provide an environment for cells which mimmicks the situation *in vivo* [27]. In this article we introduced a microfluidic approach using cell chips, hydrogel (Matrigel) and a fluorescent dye for living cells.

Breast cancer cells (MDA-MB-231) and fibrosarcoma cells (HT-1080) transfected with microRNA-299-3p (and unstimulated as negative control, respectively) were seeded in cell culture chips, which were already filled with dyed Matrigel.

Unstimulated breast cancer and fibrosarcoma cells (MDA-MB-231 and HT-1080) show an invasive behavior very similar to native metastases taken from biopsies [28]. Interestingly, like *in vivo*, the cancer cells *in vitro* are not exactly the same, but they show a hierarchical organization. A small number of cells become “leader” or “tip cells” and start to migrate into the digested matrix. Other “follower” cells, presumably attracted by chemokines from their siblings, use the migration channel created by the leaders to move into the matrix.

**Fig 4:**
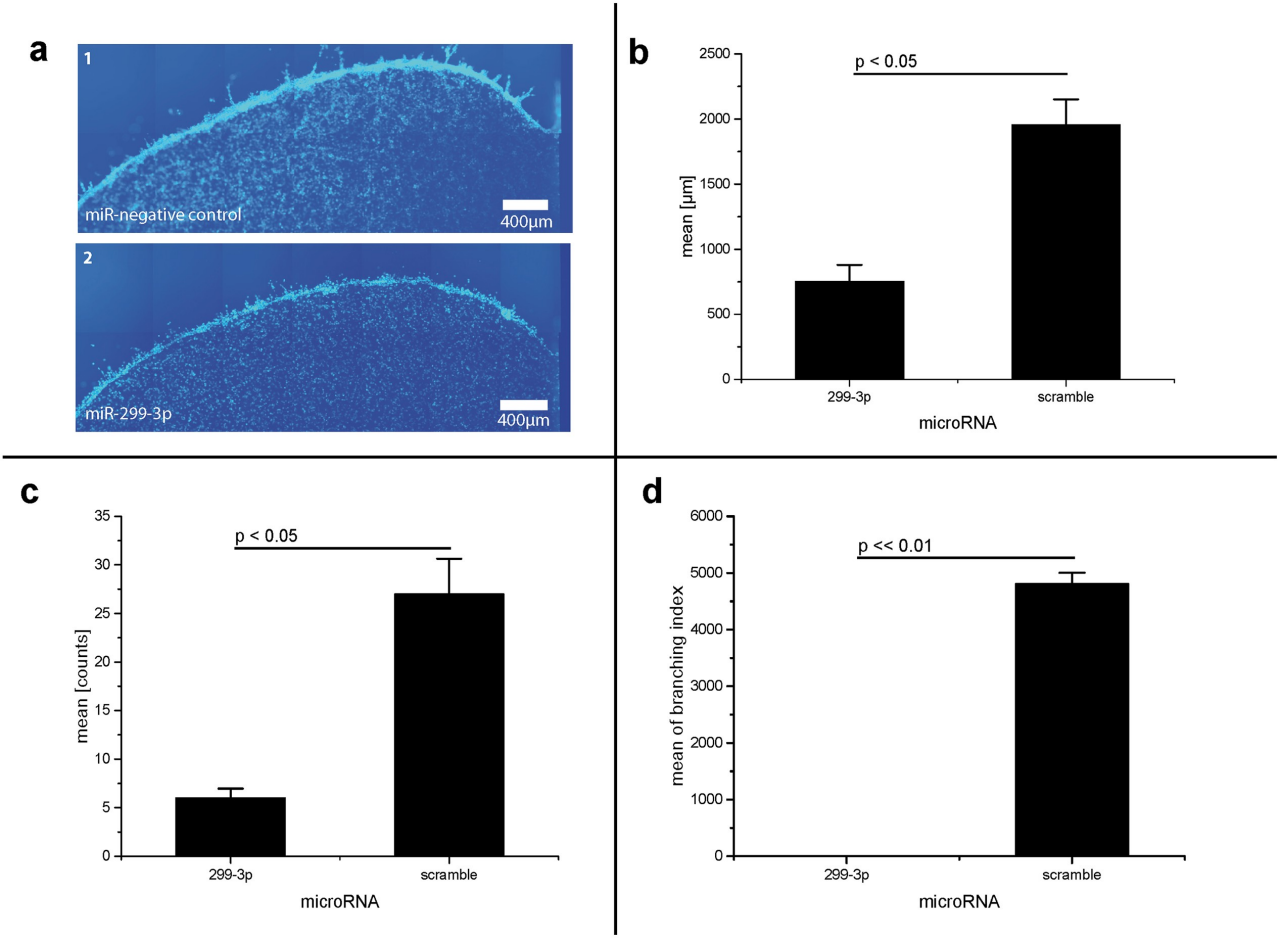
MicroRNA-299-3p suppresses invasion of fibrosarcoma cancer cells (part I). **a: Decreased invasive behavior of HT-1080 cells caused by transfection with microRNA-299-3p (fluorescence sample images)**. HT-1080-cells transfected with pre-microRNA-scramble (negative control, 1) and -299-3p (2), Cells with microRNA-299-3p show a decreased invasive behavior in relation to negative control, False colors images. Object lense: 10x. **b: MicroRNA-299-3p decreases invasion distance of HT-1080 fibrosarcoma cells**. Invasion assay, sum of invasion distance in μm. Scramble: non-human miRNA sequence (negative control). Error bars indicated as SEM. Non-parametric Kruskal-Wallis-test: p < 0.05. Post-hoc test results (Tukey) indicated in the graph. Error bars indicated as SEM, **c: MicroRNA-299-3p decreases number of invasion events of HT-1080 fibrosarcoma cells**. Invasion assay, sum of invasion events. Scramble: non-human miRNA sequence (negative control). Statistics: Error bars indicated as SEM. Non-parametric Kruskal-Wallis-test: p < 0.05. Post-hoc test results (Tukey) indicated in the graph. **d: microRNA-299-3p completely avoids branching of of HT-1080 fibrosarcoma cells**. Invasion assay: branches of invasion events. Mean of branching index, calculated by number of branched invasion events multiplicated with number of single branches in every event. Scramble: non-human miRNA sequence (negative control). Statistics: Non-parametric Wilcoxon test: p < 0.05. Post-hoc test results (Tukey) indicated in the graph.

**Fig 5:**
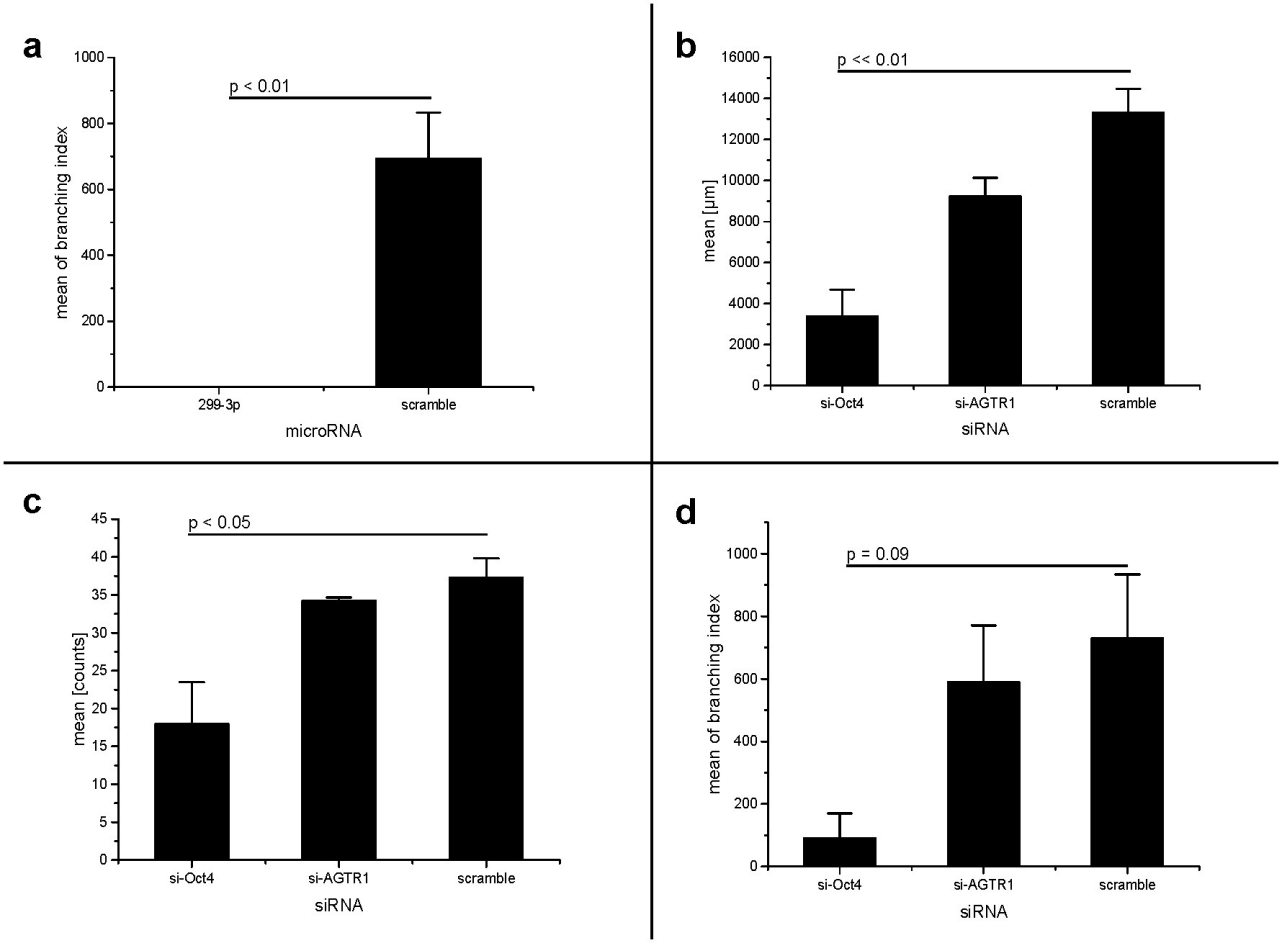
MicroRNA-299-3p suppresses invasion of mammacarcinoma cells (part II). **a: microRNA-299-3p completely avoids branching of invasive MDA-MB-231 breast cancer cells**. Invasion assay: branches of invasion events. Mean of branching index, calculated by number of branched invasion events multiplicated with number of single branches in every event. Scramble: non-human miRNA sequence (negative control). Statistics: Non-parametric Wilcoxon test: p < 0.05. Post-hoc test results (Tukey) indicated in the graph. **b: siRNA against Oct4 also decreases invasion distance of MDA-MB-231 breast cancer cells**. Invasion assay, sum of invasion distance in μm. Scramble: non-human miRNA sequence (negative control). siRNA against AGTR-1 was used as additional negative control for substance class. Statistics: Error bars indicated as SEM. Non-parametric Kruskal-Wallis-test: p << 0.01. Post-hoc test results (Tukey) indicated in the graph. **c: siRNA against Oct4 also decreases number of invasion events of MDA-MB-231 breast cancer cells**. Invasion assay, sum of invasion events. Scramble: non-human miRNA sequence (negative control). siRNA against AGTR-1 was used as additional negative control for substance class. Statistics: Error bars indicated as SEM. Non-parametric Kruskal-Wallis-test: p < 0.05. Post-hoc test result (Tukey) indicated in the graph. **d: Effect of siRNA against Oct4 on branching of invasive MDA-MB-231 breast cancer cells**. Invasion assay: branches of invasion events. Mean of branching index, calculated by number of branched invasion events multiplicated with number of single branches in every event. Scramble: non-human miRNA sequence (negative control). siRNA against AGTR-1 was used as additional negative control for substance class. Statistics: Non-parametric Kruskal-Wallis-test: p = 0.068. Post-hoc test results (Tukey) indicated in the graph.

The MDA-MB-231 and HT-1080 cells were photographed in the border area between cells and gel by fluorescence microscopy after 48 or 24 hours, respectively, (see Figs 3b and 4a). In MDA-MB-231 cells, the microRNA-299-3p transfected cells lost their invasiveness almost completely (Figs 3c and 3d, 5a and 4b to 4d).

In HT-1080 cells, the microRNA-299-3p nearly completely blocked invasion as seen above.

Some microRNAs already have been reported to have an anti-invasive effect. For instance, the microRNA-145 has been described as inhibiting proliferation and invasion of endometriosis cells. Among other genes, expression of Oct4 is down-regulated [29]. As mentioned, the constitutive blocking of Oct4 expression can reduce the invasiveness of colon cancer and bladder cancer cells [30].

**Fig 6:**
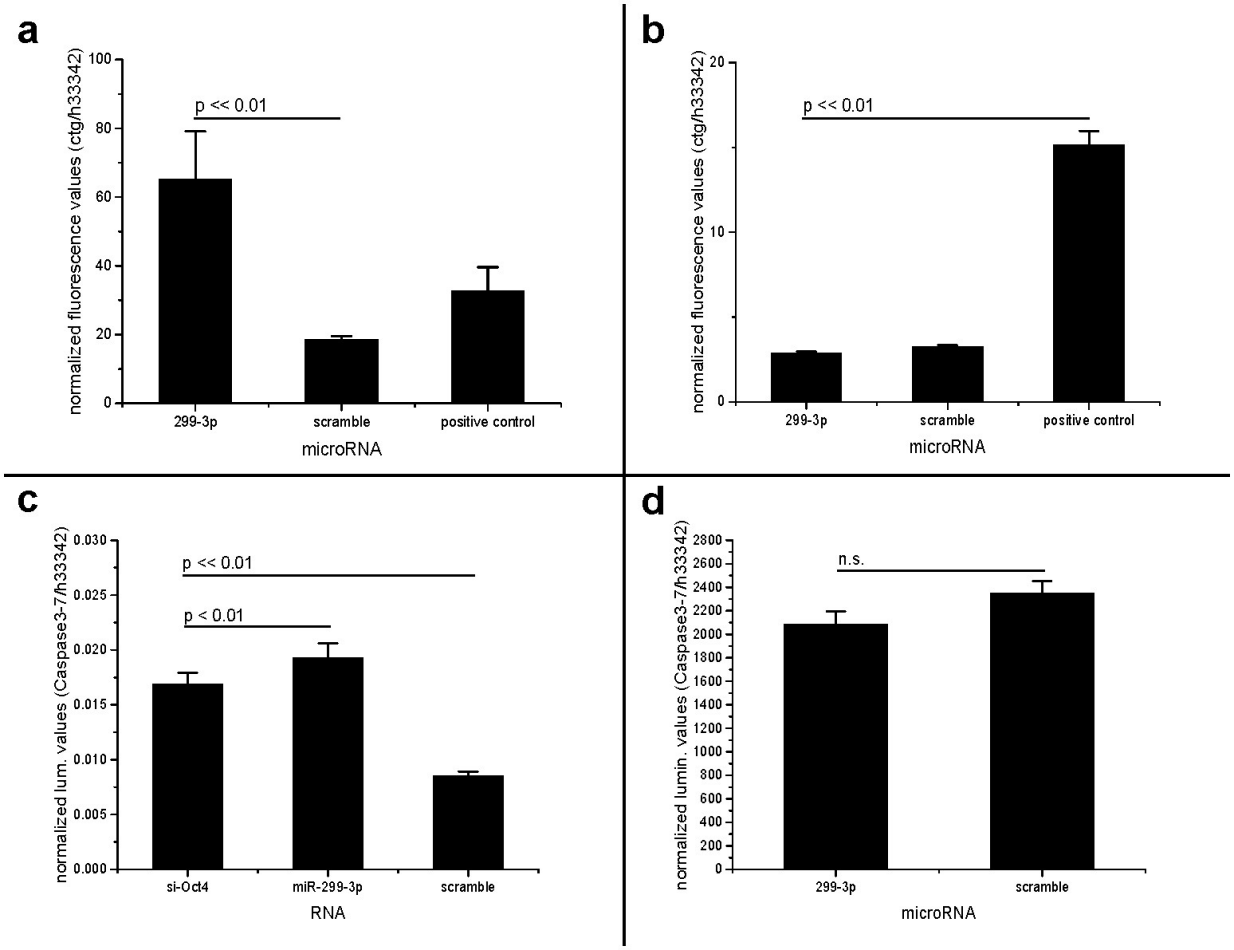
The microRNA-299-3p causes cell death in breast cancer cells, but not in non-cancer cells. **a: The microRNA-299-3p is toxic to breast cancer cells (MDA-MB-231)**. CellToxGreen cytotoxicity assay. Error bars indicated as SEM, Non-parametric Kruskal-Wallis-test: p << 0.01. Post-hoc test result (Tukey) indicated in the graph. **b: Comparison: The microRNA-299-3p is not toxic to non-cancer cells (KG-1)** CellToxGreen cytotoxicity assay. Error bars indicated as SEM, Non-parametric Kruskal-Wallis-test: p << 0.01. Post-hoc test result (Tukey) indicated in the graph. **c: The microRNA-299-3p causes apoptosis in breast cancer cells (MDA-MB-231)**. Caspase-Glo 3/7 assay. High Caspase/ Hoechst33342 quotient values correspond with high content of apoptosis enzymes. SiRNA against Oct4 serves as a control. Error bars indicated as SEM, non-parametric Kruskal-Wallis test: p < 0.01. Post-hoc test result (Tukey) indicated in the graph. **d: Comparison: The microRNA-299-3p does not cause apoptosis in non-cancer cells (KG-1)** Caspase-Glo 3/7 assay. High Caspase/ Hoechst33342 quotient values correspond with high content of apoptosis enzymes. Error bars indicated as SEM, Non-parametric Kruskal-Wallis test: p << 0.01. Post-hoc test result (Tukey) indicated in the graph: not significant.

**Fig 7:**
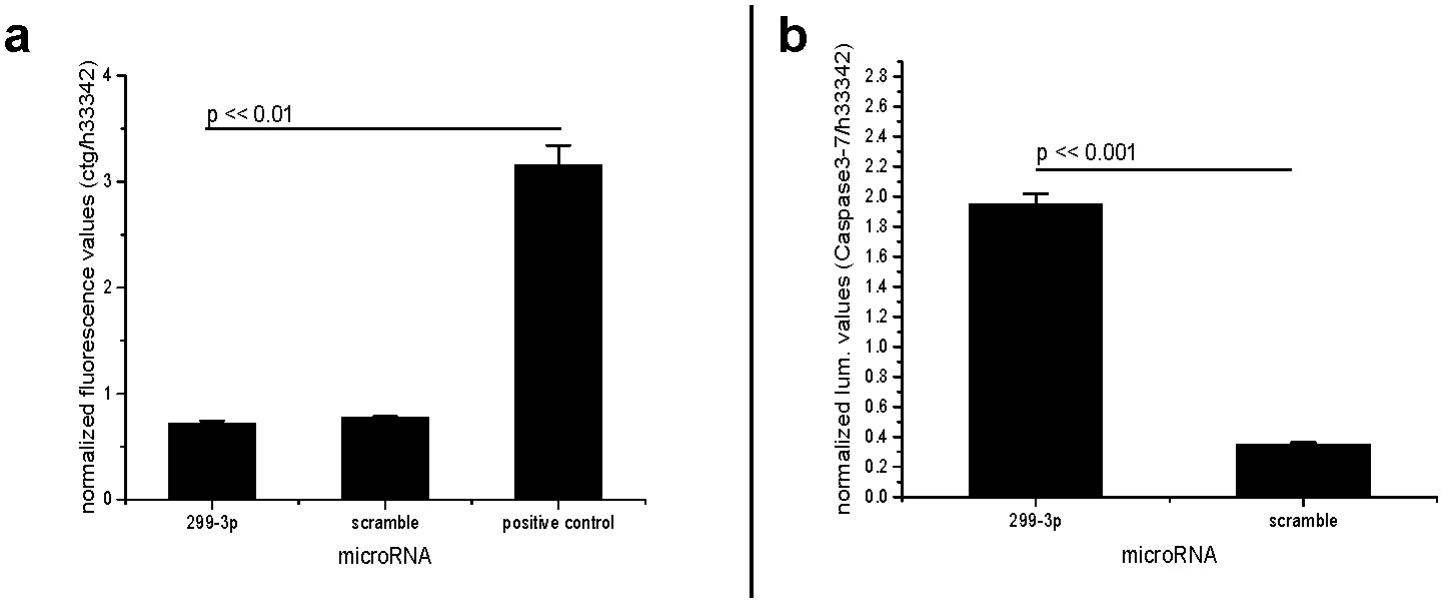
MicroRNA-299-3p is not toxic to fibrosarcoma cells, but causes apoptosis (part II). **a: The microRNA-299-3p is not toxic to HT-1080 fibrosarcoma cells**. CellToxGreen cytotoxicity assay. Error bars indicated as SEM, Non-parametric Kruskal-Wallis-test: p << 0.01. Post-hoc test result (Tukey) indicated in the graph. **b: The microRNA-299-3p causes apoptosis in HT-1080 fibrosarcoma cells**. Caspase-Glo 3/7 assay. High Caspase/ Hoechst33342 quotient values correspond with high content of apoptosis enzymes. Error bars indicated as SEM, Non-parametric Kruskal-Wallis-test: p < 0.01. Post-hoc test result (Tukey) indicated in the graph.

To analyze a potential cytotoxic and apoptotic effect of the microRNA-299-3p, transfected MDA-MB-231 and HT-1080 cells were tested with CellTox Green dye and Caspase-Glo 3/7 assay. To exclude non-stem cell gene- specific effects of miR-299-3p, we used non cancer cells (KG-1) in this assay. The miR-299-3p was toxic and clearly induced apoptosis in cancer cells; so does siRNA against Oct4 (Figs 6a, 6c and 7). This is not true for the non-cancer cells (Fig 6b and 6d); hence, it is quite probable, that miR-299-3p acts via Oct4 and related genes. However, in HT1080 cells, the miR-299-3p was not toxic. This might be due to the specific cell biology/ metabolism.

**The microRNA-299-3p** seems to be effective in inducing cell death, more specifically, apoptosis. Therefore, it can be concluded, that the reduction of invasion is just a secondary effect due to death of cells.

The comparison of the effect of miR-299-3p and siRNA against Oct4 in invasion and cytotoxic/apoptosis assays reveals the main cause of cell death: It seems that the blocked Oct4 expression leads to cell death initiation, because the application of miR-299-3p and anti-Oct4-siRNA produces similar results.

The cell death-inducing effect of other microRNAs has been described, for example for miR-146, which controls apoptosis in breast cancer cells via negative feedback loop together with NFκB [31], [32].

The microRNA-299-3p is less mentioned in the literature at present. But it has been reported to be differentially expressed in malignant mesothelioma cells [33]. Compared to murine microRNA expression patterns, the microRNA-299-3p is upregulated in human iPS and native stem cells [34]. Furthermore, it has been described to be an important factor of replicative senescence in HUVECS (*human umbilical vein endothelial cells*) [35]. These data support the view, that miR-299-3p is a factor which inhibits the expression of stem cell genes like Oct4. In human induced and native stem cells, it might initiate the differentiation process. This assumption is supported by the results of our Western blot assays, which show a decreased Oct4 protein production in miR-299-3p stimulated cells.

A close context of Oct4 and cell death is not recorded in literature. Hence, it may be concluded, that miR-299-3p may be a strong regulator of other central genes apart from Oct4, which are important factors for cell death process. This view is supported by the fact, that miR-299-3p is more toxic to breast cancer cells than anti-Oct4-siRNA.

Additionally, a bioinformatical analysis (http://www.targetscan.org) revealed some genes controlled by miR-299-3p which are connected to apoptosis. For instance, IGF-1 can promote apoptosis resistance in melanoma and pituarity cells [36], [37]. Another gene, the gene for NGFR, blocks the p53 driven anti-oncogenic processes. If these genes are negatively regulated by miR-299-3p, apoptosis process is facilitated.

In order to exclude Oct4 independent effects of microRNAs-299-3p anti-Oct4-siRNA also was used in the invasion assay. Thus, it can be concluded, that miR-299-3p not only targets the Oct4 gene transcript, but also many others. This might lead to the toxic effect we could observe in MDA-MB-231 and HT-1080 cells transfected with miR-299-3p.

## Conclusion

Our results suggest that microRNA-299-3p is an interesting target for potential clinical use. It may be able to decrease invasive behaviour of carcinoma cells; or even kill these cells by causing apoptosis.

## Supporting information

S1 Fig Anti-Oct4-siRNA downregulates Oct4 expression comparable to microRNA-299-3p. Luciferase reporter assay measuring expression of Oct4 target promoter. Data was taken over 24h. The mean of values of first and last ten minutes were taken to calculate the ratio. Scramble: non-human microRNA sequence (negative control). Error bars indicate SEM. Non-parametric Kruskal-Wallis-test: p < 0.01. Post-hoc test results (Tukey) indicated in the graph.
(TIF)

S2 Fig WebGestalt (http://www.webgestalt.org/) analysis of microRNA-299-3p target genes using KEGG database (Kyoto Encyclopedia of Genes and Genomes), in process groups (biological, molecular function, cellular component). (TIF)

S1 Table WebGestalt (http://www.webgestalt.org/) analysis. **A**: of malignant illness-related genes using KEGG database (Kyoto Encyclopedia of Genes and Genomes) which are putatively regulated by microRNA-299-3p. **B**: of putatively miR-299-3p influenced pathways using KEGG database (Kyoto Encyclopedia of Genes and Genomes).
(DOCX)

S2 Table Bioinformatical analysis of targets of human microRNA-299-3p in relation to apoptotic processes (http://www.targetscan.org). (XLSX)

S3 Table Bioinformatical analysis of microRNA target genes (http://www.targetscan.org). (XLSX)
