# Supplementary figures and images for: Human microRNA-299-3p decreases invasive behavior of cancer cells by downregulation of Oct4 expression and causes apoptosis
Source: PLoS One. 2017 Apr 20;12(4):e0174912. doi: 10.1371/journal.pone.0174912 (PMC5398498; doi:10.1371/journal.pone.0174912)

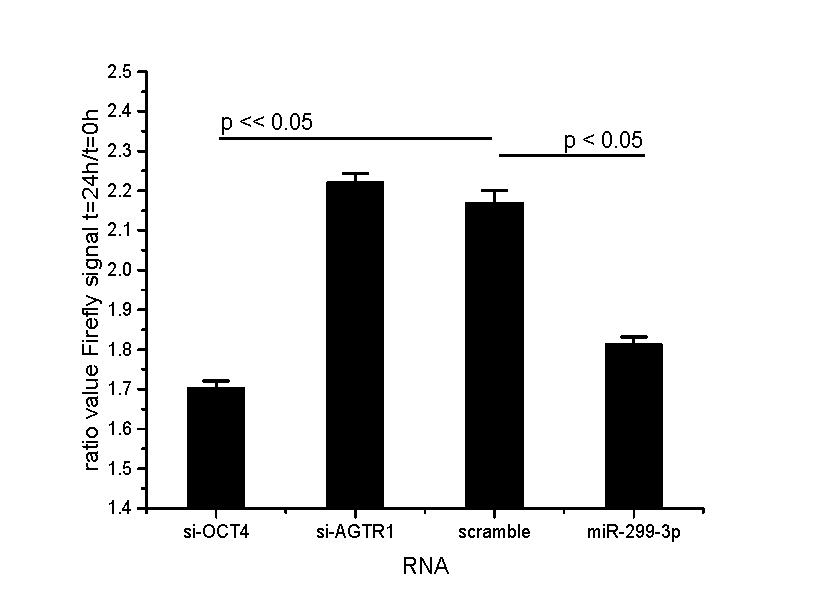

Supplement: S1 Fig — Luciferase reporter assay measuring expression of Oct4 target promoter. Data was taken over 24h. The mean of values of first and last ten minutes were taken to calculate the ratio. Scramble: non-human microRNA sequence (negative control). Error bars indicate SEM. Non-parametric Kruskal-Wallis-test: p < 0.01. Post-hoc test results (Tukey) indicated in the graph. (TIF) [file pone.0174912.s001.tif]

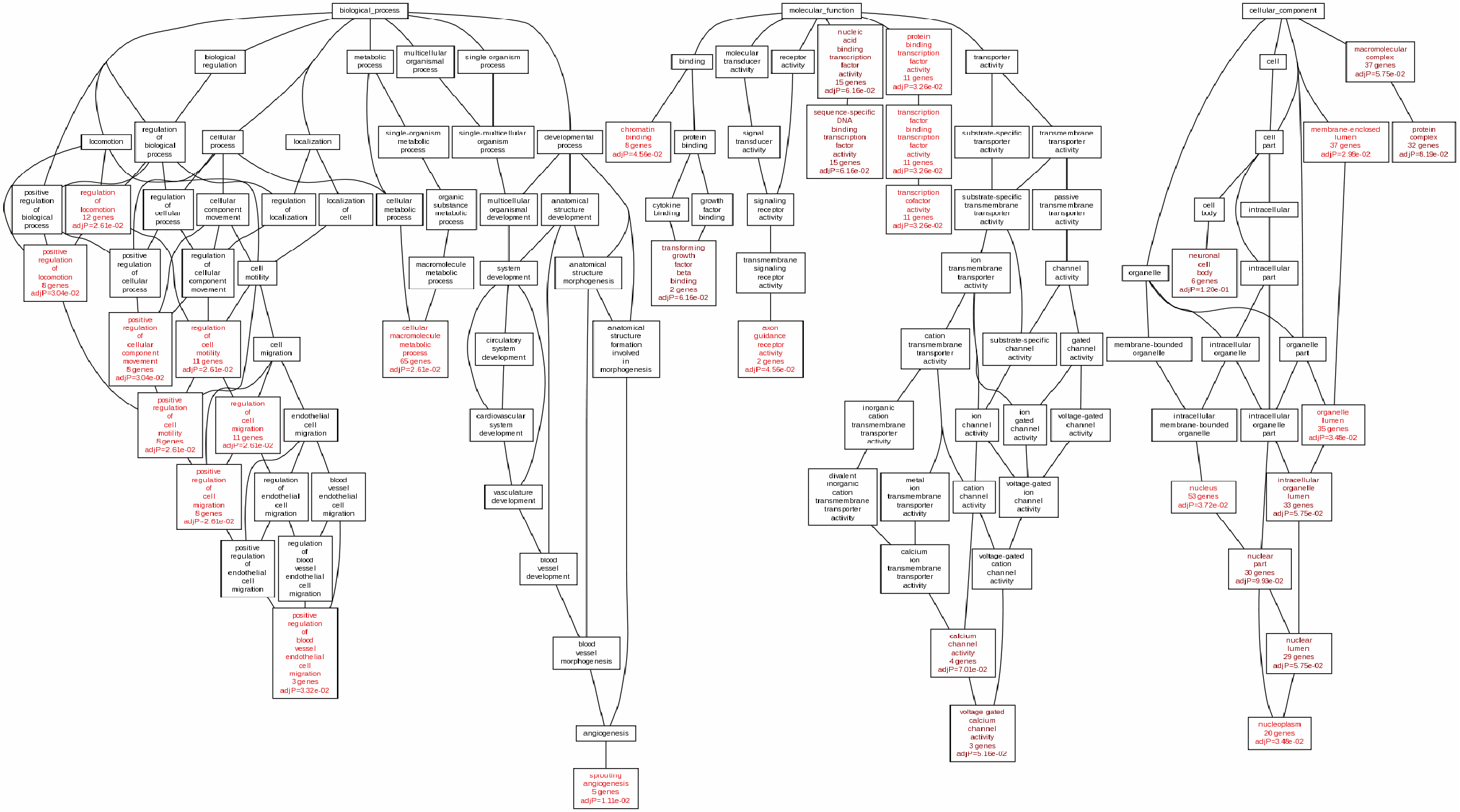

Supplement: S2 Fig — (TIF) [file pone.0174912.s002.tif]
