# Supplementary material for: Human microRNA-299-3p decreases invasive behavior of cancer cells by downregulation of Oct4 expression and causes apoptosis
Source: PLoS One. 2017 Apr 20;12(4):e0174912. doi: 10.1371/journal.pone.0174912 (PMC5398498; doi:10.1371/journal.pone.0174912)
Supplement: S1 Table — A: of malignant illness-related genes using KEGG database (Kyoto Encyclopedia of Genes and Genomes) which are putatively regulated by microRNA-299-3p. B: of putatively miR-299-3p influenced pathways using KEGG database (Kyoto Encyclopedia of Genes and Genomes). (DOCX) [file pone.0174912.s003.docx]

**WebGestalt (http://www.webgestalt.org/) analysis of malignant illness-related genes of miRNA-299-3p using KEGG database**

**Legend:**

C: the number of reference genes in the category

O: the number of genes in the gene set and also in the category

E: the expected number in the category

R: ratio of enrichment

rawP: p value from hypergeometric test

adjP: p value adjusted by the multiple test adjustment

**B: WebGestalt (http://www.webgestalt.org/) analysis of miR-299-3p influenced pathways using KEGG database (Kyoto Encyclopedia of Genes and Genomes)**
